# Supplementary material for: Psychological interventions that decrease psychological distance or challenge system justification increase motivation to exert effort to mitigate climate change
Source: Commun Psychol. 2025 Nov 5;3:148. doi: 10.1038/s44271-025-00332-4 (PMC12589137; doi:10.1038/s44271-025-00332-4)
Supplement: Supplementary file 2 — Supplemental material [file 44271_2025_332_MOESM2_ESM.pdf]

Supplementary Methods

Table S1. *Descriptions of interventions and associated theoretical frameworks, adapted from ref* [1]

Intervention	Description	References
Psychological Distance	Frames climate change as a proximal risk using examples of recent natural disasters caused by climate change in each participant's nation and prompts them to write about the climate impacts on their community.	Jones et al.[2]
System Justification	Frames climate change as threatening to the way of life to each participant's nation and makes an appeal to climate action as the patriotic response.	Feygina et al.[3]
Negative Emotions	Exposes participants to ecologically valid scientific facts regarding the impacts of climate change, framed in a "doom and gloom" style of messaging, which were drawn from different real-world news and media sources.	Chapman et al.[4]
Pluralistic Ignorance	Presents real public opinion data collected by the United Nations that show what percentage of people in each participant's country agree that climate change is a global emergency.	Geiger and Swim[5]
Future-Self Continuity	Emphasises the future self-continuity by asking each participant to project themselves into the future and write a letter addressed to themselves in the present, describing the actions they would have wanted to take regarding climate change.	Hershfield et al.[6]
Work Together Norm	Combines referencing a social norm ("a majority of people are taking steps to reduce their carbon footprint") with an invitation to "join in" and work together with fellow citizens toward this common goal.	Howe et al.[7]
Scientific Consensus	Informs participants that "99% of expert climate scientists agree that Earth is warming and climate change is happening, mainly because of human activity"	van der Linden et al.[8]<br>Rode et al.[9]
Letter to Future Generations	Emphasises how one's current actions affect future generations by asking participants to write a letter to a socially close child who will read it in 25 years when they are an adult, describing current actions toward ensuring a habitable planet.	Shrum[10]<br>Wickersham et al.[11]
Effective Collective Action	Features examples of successful collective action that have had meaningful effects on climate policies (e.g., protests) or have solved past global issues (e.g., the restoration of the ozone layer).	Goldenberg et al.[12]<br>Lizzio-Wilson et al.[13]
Dynamic Social Norms	Informs participants of how country-level norms are changing and "more and more people are becoming concerned about climate change", suggesting that people should take action.	Sparkman and Walton[14]
Binding Moral Foundations	Invokes authority (e.g., "from scientists to experts in the military, there is near universal agreement"), purity (e.g., keep our air, water, and land pure"), and ingroup-loyalty (e.g. it is the American solution") moral foundations.	Wolsko et al.[15]

Bootstrapping analysis

We simulated 1,000 datasets with the same structure and size as our real dataset. Without collecting more data, which would be impossible across multiple countries and authors, this is the best available way of establishing further substantive evidence for the strength of our results. We did this by sampling participant numbers with replacement the same number of times as the total number of participants (3,055), maintaining participants' allocations to their intervention group. We did this for both choices and *K* parameters separately. On each simulated dataset, we then calculated the average difference between the control group and each intervention condition (raw % change for choices, raw difference in *Ks*) – corresponding to the averages plotted in Figure 3 and Figure 4C respectively. We also ran the relevant mixed model on the simulated dataset and extracted the standardised effect size (odds ratio) for the cause * intervention interactions. These correspond to the model terms, with their associated p values, that we use for our conclusions. For each intervention and each outcome measure (choices and *Ks*), this generates 1000 samples with two effect size metrics: one raw and one from the model.

Computational model space

Based on all combinations of discount functions and whether parameters were charity-specific, the full model space was:

Model 1: Parabolic, 2*K*1*β*
Model 2: Linear, 2*K*1*β*
Model 3: Hyperbolic, 2*K*1*β*
Model 4: Parabolic, 2*K*2*β*
Model 5: Linear, 2*K*2*β*
Model 6: Hyperbolic, 2*K*2*β*

The linear, hyperbolic, and parabolic models were specified as follows:

Parabolic: $SV_{(t)} = R_{(t)} - \left(K * {E_{(t)}}^2\right)$ [1]
Linear: $SV_{(t)} = R_{(t)} - \left(K * E_{(t)}\right)$ [2]
Hyperbolic: $SV_{(t)} = \frac{R_{(t)}}{1 + \left(K * E_{(t)}\right)}$ [3]

The models assume that the subjective value (SV) of the offer on trial (t) is determined by the effort level (E; proportion of maximum thresholded boxes, coded as level 2, 3, 4, 5), reward level (R; credits), and the subject-specific discounting parameter (*K*), which describes the steepness of each individual's devaluation of rewards by effort. The higher the *K* value, the steeper the discount function. Note that each individual's discounting function is referenced to the SV of the baseline offer (which was always 3 credits, coded as level 1 vs. reward levels 2, 3, 4).

The *softmax* function was defined as:

$$\Pr(i) = \frac{e^{\beta.SV_i}}{e^{\beta} + e^{\beta.SV_i}} \quad [4]$$

where Pr(i) represents the probability of choosing option *i* that has a subjective value of (i), and β is the *softmax* parameter that defines the consistency (or inverse stochasticity) of each participant's choices.

Model fitting

The hierarchical MAP approach first fits the data at the individual subject level using MLE and then implements a second, higher level across the full sample. MLE at the first level provides the expectation, calculating the log-likelihood of the choices for each participant, given the model. Next, the maximum posterior probability estimate is computed using the observed choices and prior from the group-level Gaussian. Initial group-level Gaussians are uninformative priors (mean=0.1 plus noise, variance=100) then this distribution is recomputed during maximisation and the expectation and maximisation steps are iteratively repeated. We repeated these steps until the group-summed posterior likelihood converged (relative change in posterior likelihood <0.001) or the maximum number of iterations (800) was reached. To ensure accurate estimation and a plausible range of parameter values, we used appropriate transfer functions to transform estimates from Gaussian to native model space[16,17].

We identified the best fitting model (2K1β linear) based on complementary evidence from Bayesian model comparison, based on the integrated Bayesian Information Criterion (BICint; lower is better[18,19]) and the exceedance probability (XP; higher is better[20]). The XP represents the posterior probability that each model is the most likely of the model set in the population. This was calculated from random-effects analysis of the Laplace approximation of the log model evidence using the spm_BMS routine[21] from SPM8 (http://www.fil.ion.ucl.ac.uk/spm/software/spm8/). Finally, we calculated R^2 for each model as the squared median choice probability across trials and participants.

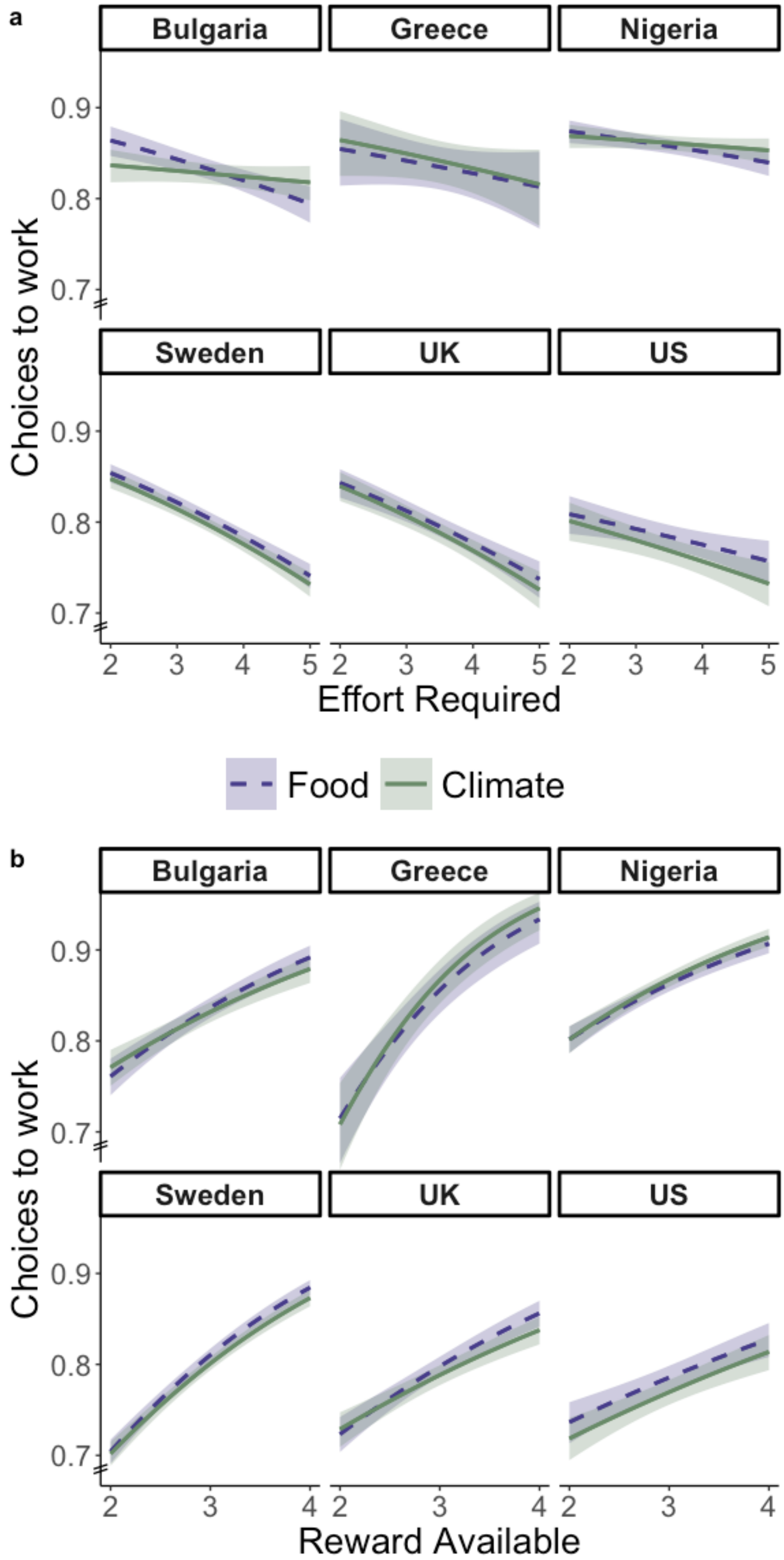

Figure S1. Effects of effort level and reward magnitude on choosing the high-effort high-reward work option replicates across countries. (a) Participants in all six countries were less willing to work when the effort required was greater and **(b)** more willing to work when the reward available was larger.

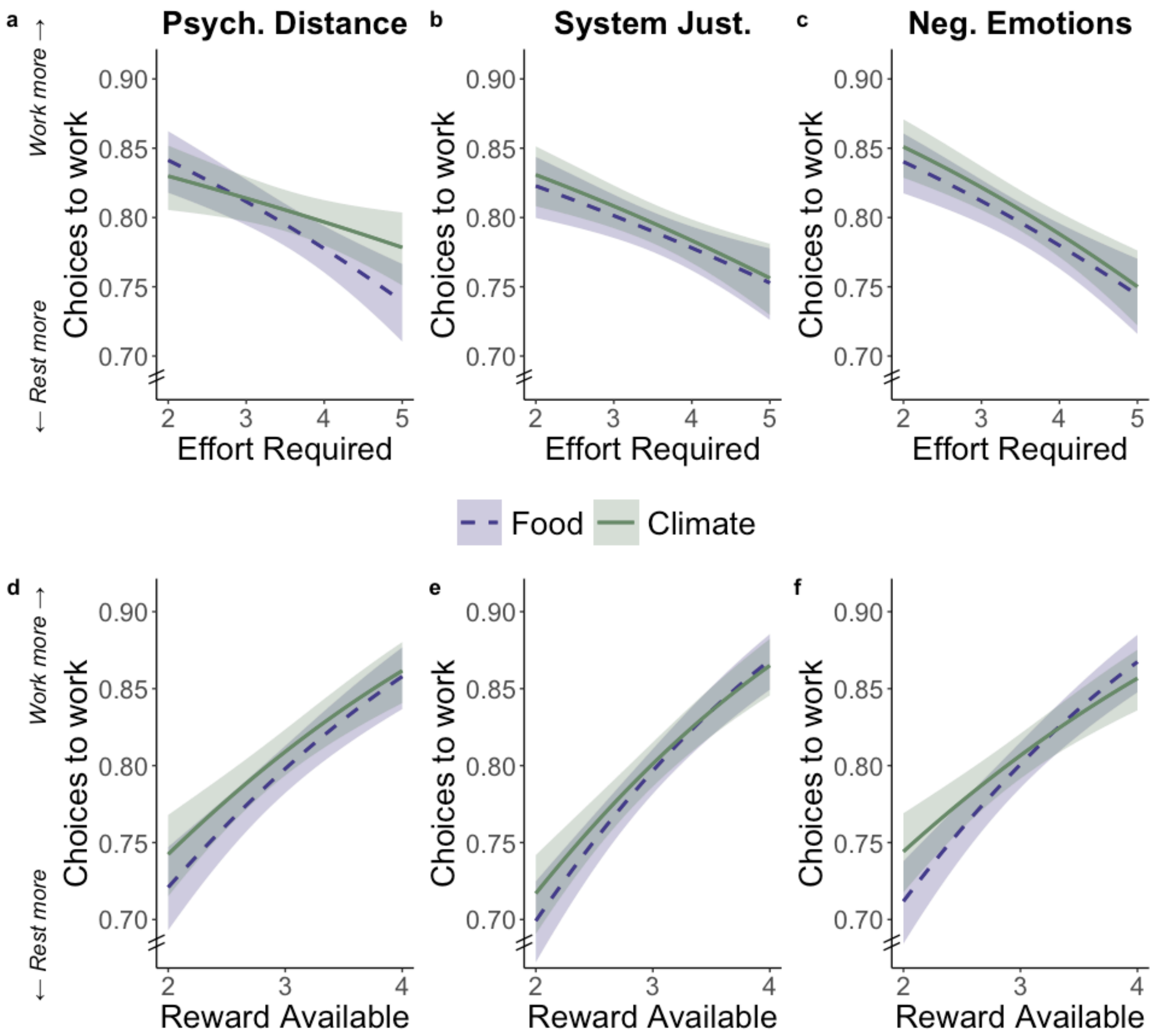

Figure S2. Effects of effort level and reward magnitude on choosing the high-effort high-reward work option following interventions that successfully increase choices for the climate: (a) Psychological Distance **(b)** System Justification and **(c)** Negative Emotions.

Table S2. *Number of participants in each intervention condition for each country*

	Bulgaria	Greece	Nigeria	Sweden	UK	US	Total
Control	42	11	55	103	42	30	283
Work Together Norm	30	8	56	80	42	26	242
Negative Emotions	42	8	51	89	36	31	257
Scientific Consensus	33	11	58	101	43	25	271
Effective Collective Action	29	11	56	99	48	28	271
System Justification	31	7	62	92	43	40	275
Psychological Distance	32	0	56	93	41	20	242
Pluralistic Ignorance	36	9	60	98	38	34	275
Letter to Future Generation	30	5	47	76	28	17	203
Dynamic Social Norms	37	9	59	89	47	26	267
Future-Self Continuity	28	6	56	67	29	25	211
Binding Moral Foundations	34	0	44	103	45	32	258
Total	404	85	660	1090	482	334	3055

Supplementary Notes

Control analyses of Work for Environmental Protection Task (WEPT)

In the model of choices that controlled for number of pages completed on the WEPT, we additionally examined the association between this control variable and willingness to choose effort in the PEET. We found that participants who completed more pages on the WEPT were also more willing to exert effort in the PEET (OR=1.73 [1.59, 1.88], p<0.001). This positive association between PEET and WEPT measures was unexpected given we found that several interventions increased willingness to work in the PEET but previous results reported that these interventions did not increase motivation to complete the WEPT, in a sample that included our participants[1]. We therefore examined intervention effects on pages completed in the WEPT using cumulative link mixed models (CLMM; see Methods) in all participants in the ICPC sample from the six countries (of 63 in total) we collected data in. As previously reported[1], no interventions significantly increased pages completed in the WEPT and several had significant negative impacts: Psychological Distance, Negative Emotions, Work-Together Norm, and Letter to Future Generation (Table S5). Therefore, differences between the two studies in how interventions increased pro-environmental effort were not due to our sample being unrepresentative or underpowered. Finally, we probed whether intervention effects would be observed when only including participants who completed the WEPT and also met pre-registered exclusion criteria for analysis of the PEET. Here, five interventions significantly *increased* the number of pages completed in the WEPT: Pluralistic Ignorance, Scientific Consensus, Collective Action, Dynamic Social Norms, and Binding Moral Foundations (Table S6). This result highlights the importance of adequate checks for attention and choice options with participants in the PEET having to decide between working or resting. We also note that controlling for time spent in the study did not impact on intervention effects in the PEET (Table S4), whereas controlling for the length of interventions also changed the effect of interventions on the WEPT results[1].

Validity of the Prosocial Effort for the Environment Task (PEET)

We additionally conducted several validity checks of the PEET to robustly establish the validity of our novel task. First, it could be argued that clicking boxes is not perceived as effortful or tiring but instead might be seen as a challenge or game. We therefore asked participants after the task to complete the NASA Task Load Index[22] to test whether they perceived the highest effort level as significantly more effortful than the lowest and compared self-reports of tiredness after the PEET compared to before it (both ratings on 0-100 scales). Results showed that the highest effort level (mean rating=58.85) was perceived as significantly harder than the lowest effort level (mean=37.36, difference d=0.81 [0.77, 0.85], p<0.001). Similarly, participants were more tired at the end (mean rating=61.25) than before the start of the PEET (mean=49.67, difference d=0.48 [0.44, 0.52], p<0.001). Next, we considered the potential role of risk avoidance as an explanation for choosing rest over work. We compared the overall success rate after choosing to work in both conditions, as if success rates were low this would suggest risk was a factor driving participant choice. Instead, we observed high success rates in both conditions (climate mean=92.32%, food mean=91.54%) meaning when people did choose to work they were able to achieve the effort they chose. Finally, we examined the possibility of a ceiling effect in the PEET. This revealed 27.33% of participants chose the work option on all of 24 PEET trials. By comparison, for the WEPT task, the proportion at ceiling (8/8 pages completed) was significantly higher (61.05%, comparison $\chi^2_{(1)}$, p<0.001).

Bootstrapping analysis

For all of the significant effects in the paper, as determined by the reported p value, across choices and *Ks*, it was the case that the distributions (95th percentile) of our 1,000 simulated datasets did not include the value corresponding to no effect. This was true for both the analysis of raw differences (null = 0, alternative hypothesis >0) and model odds ratios (null = 1, alternative hypothesis for choices <1, Supplementary Table 7; for *Ks* >1, Supplementary Table 11). In contrast, the interventions that did not show a significant effect did include the null value in their distributions (see tables below). In all cases, the medians of the simulated distributions closely reflected the effect sizes reported in the main manuscript (Figure S3 & Figure S4). Taken together, these results show the sensitivity, specificity, and precision of our existing analysis and allow us to have greater confidence that the significant effects we observe are genuine effects.

Effort discounting function

Our model comparison procedure showed the best fitting model has a linear discounting function (Table S6) whereas previous studies have suggested that physical effort is discounted parabolically and squared the effort levels in the model used to predict choices[23–25]. We also preregistered analysis with quadratic effects of effort. We therefore repeated all models of choices above with effort squared. All results replicated and we report this as a control analysis (Table S7) as the model comparison showed the linear computational model provided the best fit of the current data.

Table S3. *Generalised linear mixed-effects model (GLMM) predicting choices in the control group*

**Parameter**	**OR**	**SE**	**CI low**	**CI high**	***z***	***p***
(Intercept)	16.40	2.53	12.12	22.19	18.13	<0.001
Effort	0.70	0.07	0.58	0.85	-3.63	<0.001
Reward	1.96	0.15	1.70	2.27	9.12	<0.001
Cause (Food > Climate)	1.13	0.06	1.02	1.25	2.38	0.017

Note. OR: odds ratio, SE: standard error of the mean, CI: confidence interval

Table S4. *Generalised linear mixed-effects model (GLMM) predicting choices in the full sample*

**Parameter**	**OR**	**SE**	**CI low**	**CI high**	***z***	***p***
(Intercept)	16.34	0.82	14.81	18.03	55.56	<0.001
Effort	0.76	0.04	0.68	0.84	-5.12	<0.001
Reward	1.95	0.16	1.66	2.28	8.18	<0.001
Cause (Food > Climate)	1.03	0.02	0.99	1.06	1.54	0.12

Note. OR: odds ratio, SE: standard error of the mean, CI: confidence interval

Table S5. *GLMM comparing choices in each intervention group to the control group*

Parameter	OR	SE	CI low	CI high	*z*	*p*	*Age Gen p*	*Time p*	*WEPT p*
(Intercept)	17.73	2.63	13.26	23.71	19.39	<0.001	<0.001	<0.001	<0.001
Effort	0.76	0.04	0.68	0.84	-5.12	<0.001	<0.001	<0.001	<0.001
Reward	1.95	0.16	1.66	2.28	8.17	<0.001	<0.001	<0.001	<0.001
Cause (Food > Climate)	1.13	0.06	1.02	1.26	2.36	0.018	0.018	0.022	0.018
Work Together Norm	0.76	0.16	0.50	1.16	-1.26	0.21	0.24	0.25	0.27
Negative Emotions	0.74	0.16	0.49	1.13	-1.40	0.16	0.14	0.19	0.11
Scientific Consensus	1.01	0.21	0.67	1.52	0.05	0.96	0.77	0.84	0.72
Effective Collective Action	0.81	0.17	0.54	1.23	-0.98	0.33	0.36	0.33	0.13
System Justification	0.77	0.16	0.51	1.16	-1.25	0.21	0.23	0.32	0.18
Psychological Distance	0.77	0.16	0.50	1.17	-1.24	0.22	0.2	0.19	0.13
Pluralistic Ignorance	0.99	0.21	0.66	1.50	-0.04	0.97	0.99	0.95	0.63
Letter to Future Generation	0.97	0.22	0.62	1.52	-0.13	0.9	0.94	0.7	0.89
Dynamic Social Norms	1.28	0.27	0.84	1.94	1.16	0.25	0.26	0.22	0.47
Future-Self Continuity	1.22	0.28	0.78	1.91	0.88	0.38	0.42	0.38	0.58
Binding Moral Foundations	0.94	0.20	0.62	1.43	-0.27	0.79	0.83	0.92	0.44
Cause*Work Together Norm	0.92	0.07	0.79	1.07	-1.10	0.27	0.29	0.33	0.27
Cause*Negative Emotions	0.85	0.06	0.74	0.99	-2.10	0.036	0.036	0.0493	0.036
Cause*Scientific Consensus	0.92	0.07	0.79	1.06	-1.17	0.24	0.22	0.26	0.24
Cause*Effective Collective Action	0.93	0.07	0.80	1.07	-1.02	0.31	0.32	0.36	0.31
Cause*System Justification	0.86	0.06	0.74	1.00	-2.01	0.044	0.0498	0.0496	0.043
Cause*Psychological Distance	0.82	0.06	0.71	0.96	-2.51	0.012	0.012	0.014	0.012
Cause*Pluralistic Ignorance	0.86	0.07	0.74	1.00	-1.96	0.0498	0.0503	0.056	0.049
Cause*Letter to Future Generation	1.02	0.08	0.86	1.19	0.18	0.86	0.77	0.82	0.85
Cause*Dynamic Social Norms	0.93	0.07	0.80	1.08	-0.95	0.34	0.35	0.39	0.34
Cause*Future-Self Continuity	0.85	0.07	0.72	1.00	-1.92	0.055	0.054	0.048	0.054
Cause*Binding Moral Foundations	0.92	0.07	0.79	1.07	-1.02	0.31	0.33	0.35	0.31

Note. OR: odds ratio, SE: standard error of the mean, CI: confidence interval, WEPT: Work for Environmental Protection Task, Age Gen / Time / WEPT *p*: *p* value from control GLMM controlling for age and gender / time spend on the study before the Pro-Environmental Effort Task (PEET) / number of pages completed in the WEPT

Table S6. *Cumulative link mixed model comparing pages completed in the Work for Environmental Protection Task (WEPT) in each intervention to the control group, with and without exclusions.*

	Full ICPC sample (6 countries)					***Sample included in PEET analysis***				
**Parameter**	**b**	**SE**	**CI low**	**CI high**	***p***	**b**	**SE**	**CI low**	**CI high**	***p***
Work Together Norm	-0.25	0.07	-0.40	-0.11	<0.001	-0.08	0.14	-0.36	0.20	0.57
Negative Emotions	-0.24	0.07	-0.38	-0.09	0.00	0.22	0.14	-0.07	0.50	0.13
Scientific Consensus	0.02	0.07	-0.13	0.17	0.79	0.37	0.14	0.09	0.64	0.01
Effective Collective Action	-0.14	0.07	-0.28	0.01	0.06	0.41	0.14	0.13	0.69	0.004
System Justification	-0.04	0.07	-0.19	0.10	0.55	0.06	0.14	-0.21	0.33	0.64
Psychological Distance	-0.22	0.08	-0.37	-0.07	0.00	0.27	0.14	-0.01	0.56	0.06
Pluralistic Ignorance	-0.03	0.07	-0.17	0.12	0.72	0.41	0.14	0.13	0.70	0.004
Letter to Future Generation	-0.20	0.08	-0.36	-0.05	0.01	0.03	0.15	-0.26	0.32	0.85
Dynamic Social Norms	0.09	0.08	-0.06	0.23	0.25	0.35	0.14	0.07	0.62	0.015
Future-Self Continuity	-0.08	0.08	-0.23	0.08	0.32	0.27	0.15	-0.02	0.57	0.072
Binding Moral Foundations	0.13	0.08	-0.02	0.28	0.09	0.42	0.14	0.14	0.70	0.003

Note. SE: standard error of the mean, CI: confidence interval, ICPC: International Climate Psychology Collaboration, PEET: Pro-Environmental Effort Task

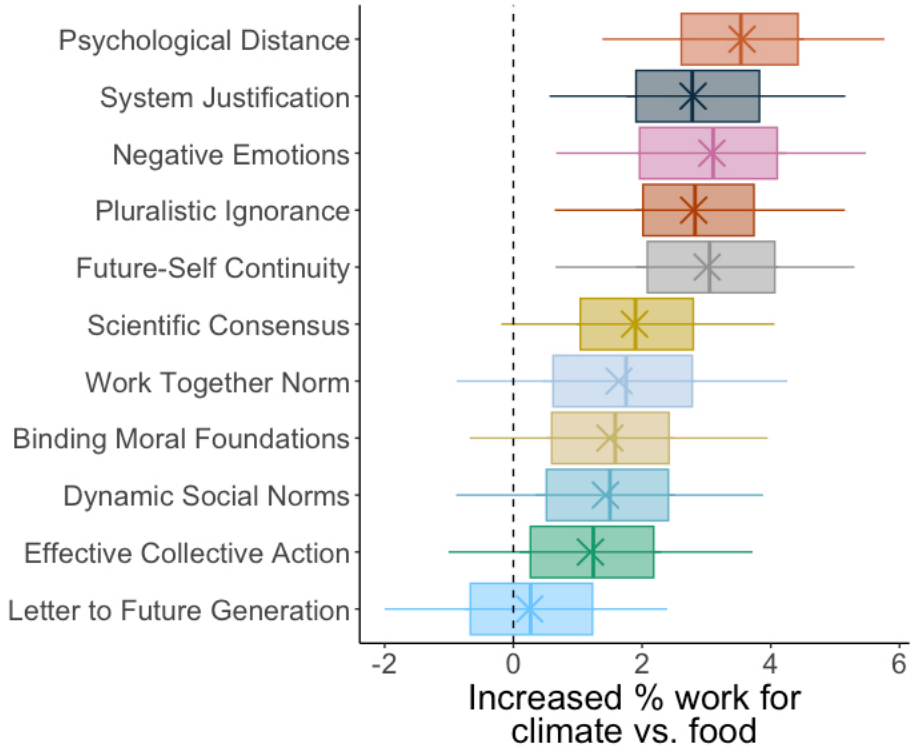

Figure S3. Simulation analysis of choices supporting effect size of successful interventions. Boxplot shows the median, lower and upper quartiles, and 5% and 95% percentiles of the simulated distributions. The X shows the effect size from the main dataset (as in Figure 3). The medians of the simulations closely capture the effect sizes in all cases. The 95% percentile does not cross 0 for the interventions that have a significant effect on choices. The upper and lower quartiles for all interventions, both significant and non-significant, could be useful for future research to provide the confidence around an expected effect size.

Table S7: Results of simulation analysis for choices

**Parameter**	**OR**	**SE**	**CI low**	**CI high**	***z***	***p***	**Raw 95%**	**Model 95%**
Cause (Food > Climate) * Work Together Norm	0.92	0.07	0.79	1.07	-1.10	0.27	-0.88	1.05
Cause (Food > Climate) * Negative Emotions	***0.85***	***0.06***	***0.74***	***0.99***	***-2.10***	***0.036***	***0.67***	***0.97***
Cause (Food > Climate) * Scientific Consensus	0.92	0.07	0.79	1.06	-1.17	0.24	-0.19	1.02
Cause (Food > Climate) * Effective Collective Action	0.93	0.07	0.80	1.07	-1.02	0.31	-1.01	1.05
Cause (Food > Climate) * System Justification	***0.86***	***0.06***	***0.74***	***1.00***	***-2.01***	***0.044***	***0.56***	***0.97***
Cause (Food > Climate) * Psychological Distance	***0.82***	***0.06***	***0.71***	***0.96***	***-2.51***	***0.012***	***1.38***	***0.93***
Cause (Food > Climate) * Pluralistic Ignorance	***0.86***	***0.07***	***0.74***	***1.00***	***-1.96***	***0.0498***	***0.64***	***0.97***
Cause (Food > Climate) * Letter to Future Generation	1.02	0.08	0.86	1.19	0.18	0.86	-2	1.16
Cause (Food > Climate) * Dynamic Social Norms	0.93	0.07	0.80	1.08	-0.95	0.34	-0.89	1.07
Cause (Food > Climate) * Future-Self Continuity	0.85	0.07	0.72	1.00	-1.92	0.055	0.65	0.98
Cause (Food > Climate) * Binding Moral Foundations	0.92	0.07	0.79	1.07	-1.02	0.31	-0.68	1.05

Note. OR: odds ratio, SE: standard error of the mean, CI: confidence interval. Columns up to *p* duplicate those from Table S5 with rows that do not correspond to interaction effects not shown for brevity. The final two columns give the results of the simulation analyses. Effect 95% corresponds to the 95% percentile of the distribution of raw differences in choice % for climate vs. food in 1,000 simulated datasets. The null hypothesis here is 0 and the effect in the real data is positive. Interventions with a significant effect are shown in bold italic and none of the 95th percentile values cross 0. Model 95% provides the 95% percentile of the distribution in odds ratios for the mixed model of choices in 1,000 simulated datasets. The null hypothesis here is 1 and the effect in the real data is below 1 (see first OR column). None of the interventions with a significant effect cross 1.

Table S8. *Model comparison*

**Model**	**LME**	**BIC_{int}**	**XP**	**R^2**
2K1β-parabolic	-35436.65	70311.16	0.24	0.75
2K1β-linear	-35292.27	69977.86	0.76	0.76
2K1β-hyperbolic	-35293.70	69921.64	0.00	0.71
2K2β-parabolic	-35915.34	71321.22	0.00	0.75
2K2β-linear	-35668.70	70786.40	0.00	0.75
2K2β-hyperbolic	-35523.41	70417.44	0.00	0.72

Note. LME: log model evidence, BIC_{int}: integrated Bayesian information criterion, XP: exceedance probability

Table S9. *GLMM of choices in each intervention to the control group with effort squared*

**Parameter**	**OR**	**SE**	**CI low**	**CI high**	***z***	***p***	***Age Gen p***	***Time p***	***WEPT p***
(Intercept)	17.72	2.64	13.23	23.72	19.30	<0.001	<0.001	<0.001	<0.001
Effort (squared)	0.76	0.04	0.68	0.85	-4.80	<0.001	<0.001	<0.001	<0.001
Reward	1.95	0.16	1.66	2.29	8.16	<0.001	<0.001	<0.001	<0.001
Cause (Food > Climate)	1.13	0.06	1.02	1.26	2.37	0.018	0.018	0.022	0.018
Work Together Norm	0.76	0.16	0.50	1.17	-1.25	0.21	0.24	0.25	0.28
Negative Emotions	0.74	0.16	0.49	1.13	-1.39	0.16	0.14	0.19	0.11
Scientific Consensus	1.01	0.21	0.67	1.53	0.05	0.96	0.77	0.84	0.72
Effective Collective Action	0.82	0.17	0.54	1.23	-0.97	0.33	0.37	0.33	0.13
System Justification	0.77	0.16	0.51	1.16	-1.25	0.21	0.24	0.31	0.18
Psychological Distance	0.77	0.17	0.50	1.17	-1.24	0.22	0.2	0.19	0.14
Pluralistic Ignorance	0.99	0.21	0.66	1.50	-0.03	0.97	1	0.95	0.63
Letter to Future Generation	0.97	0.22	0.62	1.52	-0.13	0.9	0.94	0.7	0.89
Dynamic Social Norms	1.28	0.27	0.84	1.94	1.16	0.25	0.26	0.21	0.47
Future-Self Continuity	1.22	0.28	0.78	1.91	0.88	0.38	0.42	0.38	0.58
Binding Moral Foundations	0.94	0.20	0.62	1.43	-0.27	0.79	0.83	0.92	0.44
Cause*Work Together Norm	0.92	0.07	0.79	1.07	-1.10	0.27	0.29	0.33	0.27
Cause*Negative Emotions	0.85	0.06	0.74	0.99	-2.10	0.036	0.036	0.0494	0.036
Cause*Scientific Consensus	0.92	0.07	0.79	1.06	-1.17	0.24	0.22	0.26	0.24
Cause*Effective Collective Action	0.93	0.07	0.80	1.07	-1.02	0.31	0.32	0.36	0.31
Cause*System Justification	0.86	0.06	0.74	1.00	-2.02	0.044	0.0493	0.049	0.043
Cause*Psychological Distance	0.82	0.06	0.71	0.96	-2.51	0.012	0.012	0.014	0.012
Cause*Pluralistic Ignorance	0.86	0.07	0.74	1.00	-1.97	0.049	0.0495	0.055	0.048
Cause*Letter to Future Generation	1.02	0.08	0.86	1.19	0.18	0.86	0.78	0.82	0.85
Cause*Dynamic Social Norms	0.93	0.07	0.80	1.08	-0.96	0.34	0.34	0.39	0.33
Cause*Future-Self Continuity	0.85	0.07	0.72	1.00	-1.92	0.055	0.054	0.048	0.054
Cause*Binding Moral Foundations	0.92	0.07	0.79	1.08	-1.02	0.31	0.34	0.35	0.31

Note. OR: odds ratio, SE: standard error of the mean, CI: confidence interval, WEPT: Work for Environmental Protection Task, Age Gen / Time / WEPT *p*: *p* value from control GLMM controlling for age and gender / time spend on the study before the Pro-Environmental Effort Task (PEET) / number of pages completed in the WEPT

Table S10. *GLMM comparing discounting (K) parameters in each intervention to the control group*

Parameter	OR	SE	CI low	CI high	$t_{(6082)}$	*p*	*Age Gen p*	*Time p*	*WEPT p*
(Intercept)	0.21	0.01	0.19	0.23	-31.42	<0.001	<0.001	<0.001	<0.001
Cause (Food > Climate)	0.97	0.02	0.94	1.01	-1.51	0.13	0.14	0.082	0.13
Work Together Norm	1.04	0.07	0.92	1.19	0.65	0.51	0.54	0.57	0.62
Negative Emotions	1.05	0.07	0.92	1.20	0.78	0.44	0.42	0.47	0.4
Scientific Consensus	0.99	0.06	0.87	1.12	-0.18	0.86	0.73	0.78	1
Effective Collective Action	1.04	0.07	0.92	1.19	0.66	0.51	0.56	0.51	0.34
System Justification	1.05	0.07	0.93	1.20	0.78	0.43	0.48	0.54	0.43
Psychological Distance	1.01	0.07	0.88	1.15	0.13	0.9	0.87	0.88	0.8
Pluralistic Ignorance	0.97	0.06	0.85	1.10	-0.55	0.59	0.56	0.53	0.78
Letter to Future Generation	0.99	0.07	0.86	1.13	-0.20	0.84	0.78	0.9	0.81
Dynamic Social Norms	0.96	0.06	0.85	1.10	-0.56	0.57	0.58	0.55	0.73
Future-Self Continuity	0.97	0.07	0.85	1.12	-0.37	0.71	0.76	0.71	0.84
Binding Moral Foundations	0.99	0.07	0.87	1.13	-0.15	0.88	0.84	0.84	0.85
Cause*Work Together Norm	1.02	0.02	0.99	1.06	1.27	0.2	0.18	0.26	0.2
Cause*Negative Emotions	1.04	0.02	1.00	1.07	1.95	0.051	0.044	0.07	0.0502
Cause*Scientific Consensus	1.01	0.02	0.98	1.05	0.69	0.49	0.46	0.54	0.49
Cause*Effective Collective Action	1.02	0.02	0.99	1.06	1.12	0.26	0.25	0.31	0.27
Cause*System Justification	1.05	0.02	1.01	1.08	2.64	0.008	0.012	0.01	0.008
Cause*Psychological Distance	1.06	0.02	1.03	1.10	3.42	<0.001	<0.001	<0.001	<0.001
Cause*Pluralistic Ignorance	1.04	0.02	1.01	1.08	2.30	0.022	0.021	0.027	0.021
Cause*Letter to Future Generation	1.01	0.02	0.97	1.05	0.50	0.61	0.69	0.71	0.61
Cause*Dynamic Social Norms	1.02	0.02	0.99	1.06	1.38	0.17	0.15	0.18	0.17
Cause*Future-Self Continuity	1.04	0.02	1.00	1.08	2.22	0.027	0.021	0.025	0.027
Cause*Binding Moral Foundations	1.02	0.02	0.98	1.05	0.92	0.36	0.39	0.41	0.36

Note. OR: odds ratio, SE: standard error of the mean, CI: confidence interval, WEPT: Work for Environmental Protection Task, Age Gen / Time / WEPT *p*: *p* value from control GLMM controlling for age and gender / time spend on the study before the Pro-Environmental Effort Task (PEET) / number of pages completed in the WEPT

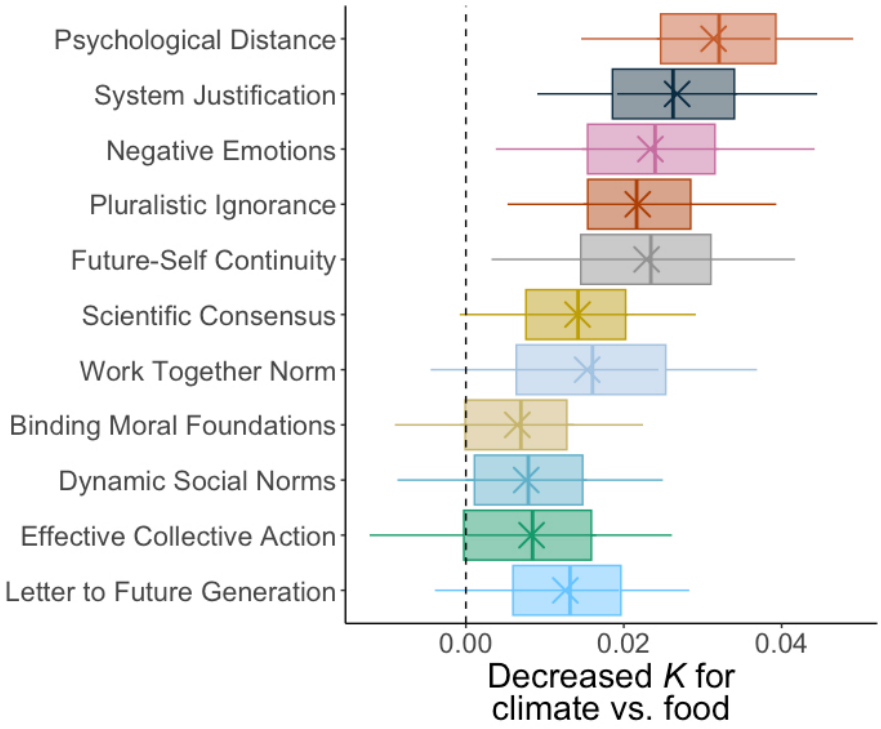

Figure S4. Simulation analysis of choices supporting effect size of successful interventions. Boxplot shows the median, lower and upper quartiles, and 5% and 95% percentiles of the simulated distributions. The X shows the effect size from the main dataset (as in Figure 4c). The medians of the simulations closely capture the effect sizes in all cases. The 95% percentile does not cross 0 for the interventions that have a significant effect on choices. The upper and lower quartiles for all interventions, both significant and non-significant, could be useful for future research to provide the confidence around an expected effect size.

Table S11: Results of simulation analysis for *K* parameters

**Parameter**	**OR**	**SE**	**CI low**	**CI high**	$t_{(6082)}$	***p***	**Raw 95%**	**Model 95%**
Cause (Food > Climate) * Work Together Norm	1.02	0.02	0.99	1.06	1.27	0.2	-0.004	0.968
Cause (Food > Climate) * Negative Emotions	1.04	0.02	1	1.07	1.95	0.051	0.004	0.997
Cause (Food > Climate) * Scientific Consensus	1.01	0.02	0.98	1.05	0.69	0.49	-0.001	0.954
Cause (Food > Climate) * Effective Collective Action	1.02	0.02	0.99	1.06	1.12	0.26	-0.012	0.964
**Cause (Food > Climate) * System Justification**	**1.05**	**0.02**	**1.01**	**1.08**	**2.64**	**0.008**	**0.009**	**1.019**
Cause (Food > Climate) * Psychological Distance	***1.06***	***0.02***	***1.03***	***1.1***	***3.42***	***<0.001***	***0.015***	***1.048***
Cause (Food > Climate) * Pluralistic Ignorance	***1.04***	***0.02***	***1.01***	***1.08***	***2.3***	***0.022***	***0.005***	***1.004***
Cause (Food > Climate) * Letter to Future Generation	1.01	0.02	0.97	1.05	0.5	0.61	-0.004	0.946
Cause (Food > Climate) * Dynamic Social Norms	1.02	0.02	0.99	1.06	1.38	0.17	-0.009	0.977
Cause (Food > Climate) * Future-Self Continuity	***1.04***	***0.02***	***1***	***1.08***	***2.22***	***0.027***	***0.003***	***1.005***
Cause (Food > Climate) * Binding Moral Foundations	1.02	0.02	0.98	1.05	0.92	0.36	-0.009	0.959

Note. OR: odds ratio, SE: standard error of the mean, CI: confidence interval. Columns up to *p* duplicate those from Table S10 with rows that do not correspond to interaction effects not shown for brevity. The final two columns give the results of the simulation analyses. Effect 95% corresponds to the 95% percentile of the distribution of raw differences in *K*s for climate vs. food in 1,000 simulated datasets. The null hypothesis here is 0 and the effect in the real data is positive. Interventions with a significant effect are shown in bold italic and none of the 95th percentile values cross 0. Model 95% provides the 95% percentile of the distribution in odds ratios for the mixed model of choices in 1,000 simulated datasets. The null hypothesis here is 1 and the effect in the real data is above 1 (see first OR column). None of the interventions with a significant effect cross 1.

Table S12. *LMM comparing choice consistency (β) parameters in each intervention to the control group*

Parameter	b	SE	CI low	CI high	$t_{(6096)}$	*p*	*Age Gen p*	*Time p*	*WEPT p*
(Intercept)	-0.01	0.06	-0.13	0.11	-0.17	0.86	0.76	0.87	0.98
Work Together Norm	-0.09	0.06	-0.21	0.03	-1.48	0.14	0.23	0.16	0.22
Negative Emotions	-0.04	0.06	-0.16	0.08	-0.64	0.52	0.51	0.53	0.45
Scientific Consensus	-0.06	0.06	-0.17	0.06	-0.94	0.35	0.49	0.36	0.20
Effective Collective Action	-0.04	0.06	-0.15	0.08	-0.62	0.54	0.67	0.6	0.28
System Justification	-0.03	0.06	-0.14	0.09	-0.47	0.64	0.86	0.73	0.64
Psychological Distance	0.00	0.06	-0.12	0.12	-0.02	0.98	0.88	0.76	0.82
Pluralistic Ignorance	-0.05	0.06	-0.16	0.07	-0.78	0.43	0.50	0.37	0.23
Letter to Future Generation	0.05	0.06	-0.08	0.18	0.76	0.45	0.32	0.43	0.43
Dynamic Social Norms	0.13	0.06	0.02	0.25	2.22	0.026	0.03	0.023	0.062
Future-Self Continuity	0.12	0.06	0.00	0.25	1.91	0.056	0.044	0.073	0.098
Binding Moral Foundations	0.02	0.06	-0.10	0.14	0.33	0.74	0.75	0.68	0.88

Note. SE: standard error of the mean, CI: confidence interval, WEPT: Work for Environmental Protection Task, Age Gen / Time / WEPT *p*: *p* value from control GLMM controlling for age and gender / time spend on the study before the Pro-Environmental Effort Task (PEET) / number of pages completed in the WEPT

Supplementary References

1. Vlasceanu, M. *et al.* Addressing climate change with behavioral science: A global intervention tournament in 63 countries. *Sci. Adv.* **10**, eadj5778 (2024).
2. Jones, C., Hine, D. W. & Marks, A. D. G. The Future is Now: Reducing Psychological Distance to Increase Public Engagement with Climate Change. *Risk Anal.* **37**, 331–341 (2017).
3. Feygina, I., Jost, J. T. & Goldsmith, R. E. System Justification, the Denial of Global Warming, and the Possibility of “System-Sanctioned Change”. *Pers. Soc. Psychol. Bull.* **36**, 326–338 (2010).
4. Chapman, D. A., Lickel, B. & Markowitz, E. M. Reassessing emotion in climate change communication. *Nat. Clim. Change* **7**, 850–852 (2017).

5. Geiger, N. & Swim, J. K. Climate of silence: Pluralistic ignorance as a barrier to climate change discussion. *J. Environ. Psychol.* **47**, 79–90 (2016).
6. Hershfield, H. E., Cohen, T. R. & Thompson, L. Short horizons and tempting situations: Lack of continuity to our future selves leads to unethical decision making and behavior. *Organ. Behav. Hum. Decis. Process.* **117**, 298–310 (2012).
7. Howe, L. C., Carr, P. B. & Walton, G. M. Normative appeals motivate people to contribute to collective action problems more when they invite people to work together toward a common goal. *J. Pers. Soc. Psychol.* **121**, 215–238 (2021).
8. van der Linden, S. L., Leiserowitz, A. A., Feinberg, G. D. & Maibach, E. W. The Scientific Consensus on Climate Change as a Gateway Belief: Experimental Evidence. *PLOS ONE* **10**, e0118489 (2015).
9. Rode, J. B., Iqbal, S., Butler, B. J. & Ditto, P. H. Using a News Article to Convey Climate Science Consensus Information. *Sci. Commun.* **43**, 651–673 (2021).
10. Shrum, T. R. The salience of future impacts and the willingness to pay for climate change mitigation: an experiment in intergenerational framing. *Clim. Change* **165**, 18 (2021).
11. Wickersham, R. H., Zaval, L., Pachana, N. A. & Smyer, M. A. The impact of place and legacy framing on climate action: A lifespan approach. *PLOS ONE* **15**, e0228963 (2020).
12. Goldenberg, A. *et al.* Testing the impact and durability of a group malleability intervention in the context of the Israeli–Palestinian conflict. *Proc. Natl. Acad. Sci.* **115**, 696–701 (2018).
13. Lizzio-Wilson, M. *et al.* How Collective-Action Failure Shapes Group Heterogeneity and Engagement in Conventional and Radical Action Over Time. *Psychol. Sci.* **32**, 519–535 (2021).
14. Sparkman, G. & Walton, G. M. Dynamic Norms Promote Sustainable Behavior, Even if It Is Counternormative. *Psychol. Sci.* **28**, 1663–1674 (2017).
15. Wolsko, C., Ariceaga, H. & Seiden, J. Red, white, and blue enough to be green: Effects of moral framing on climate change attitudes and conservation behaviors. *J. Exp. Soc. Psychol.* **65**, 7–19 (2016).
16. Daw, N. D. Trial-by-trial data analysis using computational models. in *Decision Making, Affect, and Learning: Attention and Performance XXIII* (eds. Delgado, M. R., Phelps, E. A. & Robbins, T. W.) (OUP Oxford, 2009).
17. Lockwood, P. L. & Klein-Flügge, M. C. Computational modelling of social cognition and behaviour—a reinforcement learning primer. *Soc. Cogn. Affect. Neurosci.* (2020) doi:10.1093/scan/nsaa040.
18. Wittmann, M. K. *et al.* Global reward state affects learning and activity in raphe nucleus and anterior insula in monkeys. *Nat. Commun.* **11**, 3771 (2020).
19. Huys, Q. J. M. *et al.* Disentangling the Roles of Approach, Activation and Valence in Instrumental and Pavlovian Responding. *PLOS Comput. Biol.* **7**, e1002028 (2011).
20. MacKay, D. J. C. *Information Theory, Inference and Learning Algorithms*. (Cambridge University Press, 2003).
21. Stephan, K. E., Penny, W. D., Daunizeau, J., Moran, R. J. & Friston, K. J. Bayesian Model Selection for Group Studies. *NeuroImage* **46**, 1004–1017 (2009).
22. Hart, S. G. & Staveland, L. E. Development of NASA-TLX (Task Load Index): Results of Empirical and Theoretical Research. in *Advances in Psychology* (eds. Hancock, P. A. & Meshkati, N.) vol. 52 139–183 (North-Holland, 1988).
23. Lockwood, P. L. *et al.* Prosocial apathy for helping others when effort is required. *Nat. Hum. Behav.* **1**, 1–10 (2017).
24. Lockwood, P. L. *et al.* Distinct neural representations for prosocial and self-benefiting effort. *Curr. Biol.* **32**, 4172–4185 (2022).
25. Lockwood, P. L. *et al.* Aging Increases Prosocial Motivation for Effort. *Psychol. Sci.* **32**, 668–681 (2021).
