# Supplementary material for: Psychological interventions that decrease psychological distance or challenge system justification increase motivation to exert effort to mitigate climate change
Source: Commun Psychol. 2025 Nov 5;3:148. doi: 10.1038/s44271-025-00332-4 (PMC12589137; doi:10.1038/s44271-025-00332-4)
Supplement: Supplementary file 3 — Reporting summary [file 44271_2025_332_MOESM3_ESM.pdf]

nature portfolio

Corresponding author(s): Dr Jo Cutler & Professor Patricia Lockwood

Last updated by author(s): Sep 15, 2025

Reporting Summary

Nature Portfolio wishes to improve the reproducibility of the work that we publish. This form provides structure for consistency and transparency in reporting. For further information on Nature Portfolio policies, see our Editorial Policies and the Editorial Policy Checklist.

Statistics

For all statistical analyses, confirm that the following items are present in the figure legend, table legend, main text, or Methods section.

n/a	Confirmed	
☐	☒	The exact sample size (*n*) for each experimental group/condition, given as a discrete number and unit of measurement
☐	☒	A statement on whether measurements were taken from distinct samples or whether the same sample was measured repeatedly
☐	☒	The statistical test(s) used AND whether they are one- or two-sided<br>*Only common tests should be described solely by name; describe more complex techniques in the Methods section.*
☐	☒	A description of all covariates tested
☐	☒	A description of any assumptions or corrections, such as tests of normality and adjustment for multiple comparisons
☐	☒	A full description of the statistical parameters including central tendency (e.g. means) or other basic estimates (e.g. regression coefficient) AND variation (e.g. standard deviation) or associated estimates of uncertainty (e.g. confidence intervals)
☐	☒	For null hypothesis testing, the test statistic (e.g. F, t, r) with confidence intervals, effect sizes, degrees of freedom and P value noted<br>*Give P values as exact values whenever suitable.*
☐	☒	For Bayesian analysis, information on the choice of priors and Markov chain Monte Carlo settings
☐	☒	For hierarchical and complex designs, identification of the appropriate level for tests and full reporting of outcomes
☐	☒	Estimates of effect sizes (e.g. Cohen's *d*, Pearson's *r*), indicating how they were calculated

Our web collection on statistics for biologists contains articles on many of the points above.

Software and code

Policy information about availability of computer code

Data collection	Qualtrics (website, no versions)
Data analysis	R (version 3.6.2) with R Studio (version 1.4.1106) and MATLAB (2019b, The MathWorks Inc).

For manuscripts utilizing custom algorithms or software that are central to the research but not yet described in published literature, software must be made available to editors and reviewers. We strongly encourage code deposition in a community repository (e.g. GitHub). See the Nature Portfolio guidelines for submitting code & software for further information.

Data

Policy information about availability of data

All manuscripts must include a data availability statement. This statement should provide the following information, where applicable:

- Accession codes, unique identifiers, or web links for publicly available datasets
- A description of any restrictions on data availability
- For clinical datasets or third party data, please ensure that the statement adheres to our policy

Anonymised participant-level data, source data for all figures, and all materials required to run the Pro-Environmental Effort Task are available at: https://doi.org/10.17605/osf.io/zv2tu

Research involving human participants, their data, or biological material

Policy information about studies with human participants or human data. See also policy information about sex, gender (identity/presentation), and sexual orientation and race, ethnicity and racism.

Reporting on sex and gender

Participants were recruited with the aim of being representative of the country in age and self-reported gender: total n=3,055 from Bulgaria (n=404, age 18-72, mean=41.73, 48% female), Greece (n=85: age 19-61, mean=37.05, 48% female), Nigeria (n=660, age 18-68, mean=32.27, 39% female), Sweden (n=1090, age 18-74, mean=42.84, 52% female), UK (n=482, age 18-74, mean=47.96, 56% female), and USA (n=334, age 19-74, mean=47.78, 59% female). Gender differences were not analysed as they were not part of the research question and to increase power but control analyses showed results were robust when controlling for gender (and age).

Reporting on race, ethnicity, or other socially relevant groupings

Participants were recruited from six different countries based on their residency in that country (not race or ethnicity). All analyses accounted for country-level variance through the structure of random effects in the mixed model and differences between countries were not part of the research question. Control analyses also showed effects were robust when controlling for self-reported gender (see above) and age.

Population characteristics

Total n=3,055 from Bulgaria (n=404, age 18-72, mean=41.73, 48% female), Greece (n=85: age 19-61, mean=37.05, 48% female), Nigeria (n=660, age 18-68, mean=32.27, 39% female), Sweden (n=1090, age 18-74, mean=42.84, 52% female), UK (n=482, age 18-74, mean=47.96, 56% female), and USA (n=334, age 19-74, mean=47.78, 59% female).

Recruitment

We recruited six samples (Bulgaria, Greece, Nigeria, Sweden, UK, USA) through the Marketing Science Institute recruitment company as part of the International Climate Psychology Collaboration (ICPC). Samples were recruited to be representative of gender and age distributions in each country. Participants required access to a computer to take part meaning the samples are not fully representative of people who cannot access a computer.

Ethics oversight

The study was approved by the following ethics review boards: University of Birmingham Science, Technology, Engineering and Mathematics (STEM) ethics committee (20-1897PA); The Ethics Committee of the Faculty of Business, Economics and Social Sciences of the University of Bern (232022); and University of Crete Research Ethics Committee (7875342DoPSS).

Note that full information on the approval of the study protocol must also be provided in the manuscript.

Field-specific reporting

Please select the one below that is the best fit for your research. If you are not sure, read the appropriate sections before making your selection.

☐ Life sciences ☒ Behavioural & social sciences ☐ Ecological, evolutionary & environmental sciences

For a reference copy of the document with all sections, see nature.com/documents/nr-reporting-summary-flat.pdf

Behavioural & social sciences study design

All studies must disclose on these points even when the disclosure is negative.

Study description

Quantitative experimental with a between-groups manipulation of pro-environmental psychological intervention (11 interventions + control group) and within-subjects manipulations in the main Pro-Environmental Effort Task. Participants completed a effort-based decision-making task that manipulated effort, reward and cause (climate or food charity) independently. Dependent variables measured were choices to exert effort, computational parameters from effort discounting models and additional individual difference measures completed as questionnaires.

Research sample

Total n=3,055 from Bulgaria (n=404, age 18-72, mean=41.73, 48% female), Greece (n=85: age 19-61, mean=37.05, 48% female), Nigeria (n=660, age 18-68, mean=32.27, 39% female), Sweden (n=1090, age 18-74, mean=42.84, 52% female), UK (n=482, age 18-74, mean=47.96, 56% female), and USA (n=334, age 19-74, mean=47.78, 59% female). Each sample recruited with the aim of being representative on age and gender distributions in the country. Countries to sample determined by the research team's access to data collection in the country and ability to produce the materials in the relevant language.

Sampling strategy

Stratified sampling with the aim of collecting representative samples on age and gender for each country. Sample size determined by an requirement to collect 500 participants (before exclusion criteria applied) in each country as part of the ICPC study and the aim to collect as many participants as possible to maximise power for individual differences analyses. Sample sizes were not determined using a statistical technique but were preregistered. The numbers of eligible participants who reached the novel PEET experiment [against preregistered recruitment aims] were – Bulgaria: 727 [500], Greece: 146 [500; technical problem outlined in manuscript], Nigeria: 1346 [1000], Sweden: 2056 [1500], UK: 856 [500], USA: 735 [500]. After exclusion criteria these numbers were Bulgaria: 404, Greece: 85, Nigeria: 660, Sweden: 1090, UK: 482, USA: 334.

Data collection

Data were collected on a computer. Participants completed a single online testing session with no experimenter present during study completion. Other people could have been present but we do not have this information due to the online nature of the study.

Timing

November - December 2022.

Data exclusions	Participants who failed an attention check at the very start of the survey were immediately excluded and replaced with another participant. The following numbers of unique participants completed the first, ICPC part of the study: Bulgaria: 792, Greece: 827, Nigeria: 1528, Sweden: 2502, UK: 964, USA: 880. Of these, participants who failed a second attention check later in the study or did not correctly complete the WEPT demo were excluded by the ICPC based on preregistered criteria (Bulgaria: 20, Greece: 149, Nigeria: 53, Sweden: 147, UK: 23, USA: 58). Unfortunately, a technical issue with the Greek version of the survey meant 532 participants were excluded as information about the nature of the study was visible before the PEET. The numbers of eligible participants who reached the novel PEET experiment [against preregistered recruitment aims] were – Bulgaria: 727 [500], Greece: 146 [500], Nigeria: 1346 [1000], Sweden: 2056 [1500], UK: 856 [500], USA: 735 [500].<br><br>After receiving instructions about the PEET, participants completed two comprehension questions. If answered incorrectly, they saw reminders of the key aspects of the task and answered the questions again. As preregistered, we excluded participants who answered both questions incorrectly on the second attempt (Bulgaria: 82, Greece: 15, Nigeria: 191, Sweden: 305, UK: 145, USA: 149). We also excluded participants who missed more than 20% of trials in the PEET, in-line with our preregistration. While this resulted in a relatively large number of exclusions (Bulgaria: 241, Greece: 46, Nigeria: 495, Sweden: 661, UK: 229, USA: 252), it is important to ensure enough trials for analysis and because missing multiple trials could indicate a lack of engagement with the task.
Non-participation	Participants were considered as dropping out of the PEET experiment if they missed more than 20% of trials. These numbers were Bulgaria: 241, Greece: 46, Nigeria: 495, Sweden: 661, UK: 229, USA: 252. We could not record the reason for not continuing participation. As recruitment was administered by the Marketing Science Institute and the first part of the study including inclusion criteria for continuing past the first attention check was applied by the ICPC core team, we do not have numbers of participants or reasons for initial non-participation.
Randomization	Participants were randomly allocated to one of the 11 intervention conditions or the control condition using randomisation in Qualtrics.

Reporting for specific materials, systems and methods

We require information from authors about some types of materials, experimental systems and methods used in many studies. Here, indicate whether each material, system or method listed is relevant to your study. If you are not sure if a list item applies to your research, read the appropriate section before selecting a response.

Materials & experimental systems

n/a	Involved in the study
☒	☐ Antibodies
☒	☐ Eukaryotic cell lines
☒	☐ Palaeontology and archaeology
☒	☐ Animals and other organisms
☒	☐ Clinical data
☒	☐ Dual use research of concern
☒	☐ Plants

Methods

n/a	Involved in the study
☒	☐ ChIP-seq
☒	☐ Flow cytometry
☒	☐ MRI-based neuroimaging

Plants

Seed stocks	*Report on the source of all seed stocks or other plant material used. If applicable, state the seed stock centre and catalogue number. If plant specimens were collected from the field, describe the collection location, date and sampling procedures.*
Novel plant genotypes	*Describe the methods by which all novel plant genotypes were produced. This includes those generated by transgenic approaches, gene editing, chemical/radiation-based mutagenesis and hybridization. For transgenic lines, describe the transformation method, the number of independent lines analyzed and the generation upon which experiments were performed. For gene-edited lines, describe the editor used, the endogenous sequence targeted for editing, the targeting guide RNA sequence (if applicable) and how the editor was applied.*
Authentication	*Describe any authentication procedures for each seed stock used or novel genotype generated. Describe any experiments used to assess the effect of a mutation and, where applicable, how potential secondary effects (e.g. second site T-DNA insertions, mosiacism, off-target gene editing) were examined.*
